# Maternal vaccination against pertussis as part of the national immunization program: a qualitative evaluation among obstetric care providers one year after the implementation in December 2019

**DOI:** 10.1186/s12913-023-09274-1

**Published:** 2023-03-30

**Authors:** Maarten M. Immink, Kim van Zoonen, Nina M. Jager, Anne J.M. Pluijmaekers, Hester E. de Melker, Nicoline A.T. van der Maas, Mireille N. Bekker

**Affiliations:** 1 Centre for Infectious Disease Control, National Institute for Public Health and the Environment (RIVM), Antonie van Leeuwenhoeklaan 9, Bilthoven, 3720 MA The Netherlands; 2 Department of Obstetrics, Wilhelmina Children’s Hospital, University Medical Center Utrecht, and Utrecht University, Utrecht, the Netherlands

**Keywords:** Pertussis, Maternal vaccination, Obstetrics, Midwives, Gynecologists, Interviews.

## Abstract

**Background:**

Immunization of pregnant women with a tetanus-diphtheria-and-acellular-pertussis (Tdap) vaccine is an effective and safe way to protect infants from pertussis before their primary vaccinations. Vaccine uptake among pregnant women is influenced by their care providers’ attitudes toward maternal vaccination. This qualitative study aimed to evaluate the implementation of the maternal Tdap vaccination under the National Immunization Program of the Netherlands from the perspective of obstetric care providers.

**Methods:**

In this qualitative and explorative study, we conducted in-depth interviews by telephone with obstetric care providers who were selected from a pool of respondents (convenience sampling) to a questionnaire in a previous study. The interviews were based on a semi-structured interview guide that covered three aspects of the implementation strategy: providers’ overall experience with the implementation of maternal Tdap vaccination in the Netherlands; implementation logistics and counseling, and pregnant women referrals to municipal Youth Healthcare Centers. The interviews were recorded, pseudonymized and transcribed verbatim. Transcripts were analyzed according to the Thematic Analysis approach by two researchers independently in two phases of iterative coding, categorizing, reviewing and redefining until ultimately, emergent themes regarding maternal Tdap vaccination implementation were identified.

**Results:**

Interviews with 11 midwives and 5 OB-GYN physicians yielded 5 major themes regarding the Tdap vaccination implementation strategy: challenges throughout the implementation process, views on maternal Tdap vaccination, general versus tailored counseling, provider responsibilities in vaccine promotion, and impact of materials for information delivery. Participants indicated that to improve provider attitudes toward Tdap vaccination, its implementation requires clear and transparent information about what is entailed, i.e., what is expected from obstetric care providers, how they can obtain information, and when their actions must be initiated. Participants demanded involvement throughout the implementation planning process. They preferred tailored communication with pregnant women over a generalized approach.

**Conclusion:**

This study emphasized the importance of involving all relevant healthcare professionals in planning the implementation of maternal Tdap vaccination. Possible barriers perceived by these professionals should be taken into account in order to improve their attitudes toward vaccination, thus to increase uptake among pregnant women.

**Supplementary Information:**

The online version contains supplementary material available at 10.1186/s12913-023-09274-1.

## Background

Pertussis is a highly contagious respiratory disease, caused mainly by the bacterium *Bordetella pertussis*. Especially young unvaccinated infants (< 6 months of age) are at risk of developing severe disease, resulting into hospitalization and sometimes death [1, 2]. *B. pertussis* is known to circulate across all age groups in many countries, including the Netherlands, despite high children’s vaccination coverage. Hence, the disease is readily transmitted by infected persons showing no typical clinical symptoms, as symptoms generally manifest milder and less typical in older children and adults, but these age groups may be a source of transmission to young infants [3–6]. A recent serosurveillance study estimated that yearly approximately 5.9% of all residents older than 7 years in the Netherlands was recently infected by *B. pertussis*, whether or not showing any disease symptoms [7]. Confirmed pertussis incidence rates in all age groups show epidemic peaks every 3 to 4 years, with infants under 5 months of age having the highest incidence ranging from 64 to 222 per 100,000 each year in the decade before the COVID-19 lockdown periods [5, 8].

Before receiving their primary vaccinations, infants depend on maternal antibodies for protection against infectious diseases. These antibodies are actively passed to infants during pregnancy through placental IgG antibody transfer [9]. Maternal vaccination against pertussis enhances the immunological protection that infants receive from their mother [10, 11]. Therefore, the Dutch Health Council advised in 2015 that vaccination against tetanus, diphtheria and acellular pertussis (Tdap) should be offered to pregnant women in the Netherlands. Initially, women could obtain the vaccine at their own expense at their general practitioner, midwife or gynecologist, or municipal healthcare center. Vaccine uptake increased rapidly in 2018 and 2019, with vaccine coverages of approximately 13% and 26%, respectively [12]. Ultimately since December 2019, the vaccination has been included in the National Immunization Program (NIP) of the Netherlands, making it available to pregnant women free of charge [13, 14].

Vaccine implementation was coordinated by the National Institute for Public Health and the Environment (RIVM) in close collaboration with representatives from Dutch professional organizations for obstetricians, midwives, and youth public healthcare physicians and nurses [14–19]. The implementation included the development of guidelines for the maternal Tdap vaccination, describing tasks for all professionals involved in this maternal vaccination program and practical information. It was decided that obstetric care providers would make pregnant women aware of the maternal Tdap vaccination well before 22 weeks of gestation, and hand out an information packet that consists of a letter and a leaflet about maternal Tdap immunization. Women would then be referred to Youth Healthcare Centers to receive the vaccination and more counseling if needed. The vaccination is offered from 22 weeks of gestation, i.e. the earliest opportunity for women get vaccinated. Nowadays, maternal vaccine coverage in the Netherlands ranges around 70% [20, 21].

Previous research has shown that the attitude of obstetric care providers toward maternal Tdap vaccination greatly affects its acceptance by pregnant women [22, 23]. Although vaccination in general is broadly supported by the public, there is some hesitancy and opposition, especially to vaccination during pregnancy [24, 25]. A well-organized implementation strategy provides a clear definition of responsibilities as well as practical tools and repeated training to enable obstetric care providers to facilitate information delivery to pregnant women [26, 27]. This qualitative study aimed to evaluate the implementation of maternal Tdap vaccination in the Netherlands from the perspective of obstetric care providers, its planning, guidelines, and the first few months of availability through NIP, and investigate how the implementation reflected their attitudes toward maternal vaccination. In addition, we explored possible improvements for future implementations of vaccinations during pregnancy.

## Materials and methods

### Study design

This qualitative and explorative study was performed according to a phenomenological approach in order to identify (novel) themes that may be specific for the unique Dutch situation for offering the maternal Tdap vaccination.

### Selection of study participants

Obstetric care providers were selected from a pool of respondents to a prior questionnaire-study of maternal Tdap vaccination [28]. They were approached for the current study if they had indicated willingness to be contacted for further qualitative research. 852 midwives and 201 gynecologist or OB-GYN residents responded to the initial questionnaire that was sent to a nationwide group of obstetric care providers from all geographical areas within our country. Convenience sampling was performed within those who stated willingness to participate in further research (23% (n = 194) and 15% (n = 31), respectively). Among those, antenatal care providers were selected and invited for study participation, assuming that both disciplines would be included in our eventual study population. No further inclusion criteria or restrictions were imposed for participation, except for that the person had to provide obstetric care at the time of inclusion, which was already mandated for completion of the questionnaire. Contact details for inviting study participants were stored in a safe environment separate from the database that was analyzed. Further details on procedures and results of the previous study were reported elsewhere [28].

The study was performed in accordance with the Declaration of Helsinki. Medical ethical approval was not necessary as this study was considered ‘non-interventional’ by the Medical Research Ethics Committee of Utrecht under reference number 20–601/C.

### Data collection

We conducted individual in-depth interviews by telephone, as the study was conducted during COVID-19 lockdown periods. The interviewer (NJ) was a female master student under guidance (by MI) of the Centre for Infectious Disease Control of the RIVM. Prior to conducting the interviews, there was no relationship between the interviewer and the participants. Pilot interviews were held with three (non-)obstetric care related healthcare professionals. Verbal informed consent had been obtained from all the participants before the start of each interview. The interviews were based on a semi-structured interview guide with open-ended questions (Table 1) regarding three aspects of Tdap implementation: (1) overall experience with the implementation of maternal Tdap vaccination in the Netherlands; (2) implementation logistics and counseling; and (3) pregnant woman referrals to Youth Healthcare Centers. It was developed based on the current knowledge of attitudes toward maternal vaccination among obstetric care providers and their effects on maternal vaccine uptake, and the results of the previous questionnaire study, unique for the Dutch Tdap vaccination implementation, and evaluated in consultation with several experts in the field of obstetrics, infectious diseases, and epidemiology [22, 27–36]. The interview guide was evaluated after every four interviews. The interviews were recorded and transcribed verbatim and fieldnotes imbedded in transcripts. For confidentiality, we assigned study numbers to all transcripts, whereafter the transcripts were moved to a safe environment separately from the databases on the questionnaire. Only three researchers (NJ, MI and KvZ) had access to the transcripts, of whom only NJ was aware which persons had been included in the study. All transcripts have been individually discussed between the interviewer and one analyzer (MI).

### Data analysis

MAXQDA qualitative analysis software version 20.0.7. was used for analysis of results. Transcripts were analyzed according to the Thematic Analysis approach [37, 38]. Two researchers (MI and KvZ) independently analyzed the transcripts in two phases to identify emergent themes systematically. The first phase of analysis consisted of coding of the transcripts, with MI coding all transcripts and KvZ coding six randomly selected transcripts. Before proceeding to the next phase, they discussed discrepancies in coded segments until consensus was reached. In the second phase, all coded segments were iteratively categorized by MI; and by KvZ for the same six she coded in the first phase. Potential themes were identified based on the categorized codes, then reviewed and redefined against the dataset to generate final themes that were relevant regarding the implementation. These are presented in the results section with verbatim quotations from the transcripts in Dutch that were translated to English by the researchers.

Throughout this study, we aimed to follow the trustworthiness criteria from Lincoln and Guba, i.e. credibility, transferability, dependability confirmability, to ensure the rigor of the results [39]. Only the transferability criterion could not be well-embedded in our study since basic demographics from the questionnaire data were unavailable.

## Results

### Interviews and themes

From March until May 2021, 16 interviews were conducted. Study participants consisted of 11 midwives, of whom 9 provided primary care (henceforth called primary care midwives) and 2 provided secondary care (secondary care midwives), and 5 physicians, of whom 4 were gynecologists (2 working in secondary care and 2 in tertiary care) and 1 was an OB-GYN resident. All participants agreed to be interviewed for this study following the initial invitation. The interviews lasted between 25 and 35 min, with one outlier of 10 min. After the fourth round of evaluating the interview guide - for which no substantive updates were necessary -, corresponding with interviews [13–16], we decided that data saturation had been reached.

Five major themes regarding the implementation emerged: (1) challenges throughout the implementation process; (2) views on maternal Tdap vaccination; (3) general versus tailored counseling; (4) provider responsibilities in vaccine promotion; and (5) impact of materials for information delivery. Table 1 indicates how the aspects of the interview guide related to the final themes. Corresponding categories, subcategories and example codes have been provided in the Supplementary table for reproducibility of the study.

**Table 1:** Interview guide

Aspect	Question
Overall experiences as to the implementation of maternal Tdap vaccination in the Netherlands	What is your general opinion about maternal Tdap vaccination?^2^
	How did you experience the implementation of the maternal Tdap vaccination?^1^
	How would you have led the implementation of the maternal Tdap vaccination, based on your current knowledge?^1,3^
	How could a future implementation of a maternal vaccination be improved?^1,3,4,5^
Implementation logistics and counseling	What does an average conversation about Tdap vaccination with a pregnant woman look like?^2,3,4,5^
	How do conversations about Tdap vaccination with pregnant women differ?^3,4,5^
	Does - and how does - the subject maternal Tdap vaccination return later throughout pregnancy?^3,4,5^
	What information materials do you use for informing pregnant women about the maternal Tdap vaccination?^5^
	What do you think of the information materials that you use for information delivery?^5^
Pregnant woman referrals to Youth Healthcare Centers	How would you describe your collaboration with Youth Healthcare Centers?^1,3,4^
	What is the added value of being notified by Youth Healthcare Centers that one of your clients/patients has received maternal Tdap vaccination?^1,4^
	How would you describe your role regarding the maternal Tdap vaccination?^3,4,5^
	How do you regard your role in relation to the role of Youth Healthcare Centers?^1,3,4,5^

Superscripted numbers indicate how the responses to questions from the interview guide predominantly related to the corresponding themes as presented in the results section: ^1^challenges throughout the implementation process; ^2^views on maternal Tdap vaccination; ^3^general versus tailored counseling; ^4^provider responsibilities in vaccine promotion; ^5^impact of materials for information delivery.

#### Challenges throughout the implementation process

Participants generally agreed that the implementation required a concrete description of what Tdap vaccination as part of the NIP entails: what is expected from obstetric care providers, how they can obtain information, and when their actions must be initiated. The information they received about what actions to take during counseling, as well as training sessions and an e-learning that was made available, were highly appreciated by the participants, and increased their confidence when informing pregnant women about maternal Tdap vaccination.

Participants found it unfortunate that after the Health Council advised maternal Tdap vaccination, four years elapsed before it was available free under the NIP. In this interval, local initiatives arose to provide the vaccination to pregnant women at their own cost. The logistics that had been put in place for that process had to be reorganized after NIP included the maternal Tdap vaccination.

*“Initially, we [midwives] worked together with general practitioners during the implementation [prior to inclusion within the NIP]. The GP would provide and inject it [the vaccine]. And actually, only a year later this was picked up by municipal healthcare services [Youth Healthcare Centers].” [interview 12, primary care midwife]*.

Once the vaccination was included under the NIP, participants needed a short period to get used to new procedures, after which execution became easier.

Some decisions made by policy makers were not fully supported nor well understood by all our participants. They argued that there were insufficient opportunities for providing input by healthcare providers during the planning process. Notably, many were unaware that representatives of their professional organizations had been involved throughout this process.

*“It is very unclear to us [midwives] whether the KNOV [Royal Dutch Organization of Midwives], for example, was included in the meetings [for guideline development]. How did that go? What was the reason to ultimately decide for the Youth Healthcare Centers [to administer the vaccine]?” [interview 15, primary care midwife]*.

Several participants (predominantly primary care midwives) argued that vaccine uptake would have been higher if the whole process had been centralized and executed by the obstetric care provider. According to some, the current strategy of referring pregnant women to Youth Healthcare Centers for vaccination is an extra hurdle for the women.

*“Pregnant women have to arrange it [obtaining the vaccine] themselves. They, themselves – especially if it concerns a first child – have to go to a new institution. This led to – well – that’s a barrier after all. [interview 4, primary care midwife]*

On the contrary, other participants (predominantly gynecologists) argued that the guidelines could be easily maintained and were the reason for the (participant-reported) high vaccination coverage. Moreover, the implemented strategy provided advantages that would have been missed if both information delivery and vaccination had been centralized at obstetric care providers.

*“The nurse who would usually initiate postpartum care now actually starts [informing about postpartum care] before that. So that they look at what kind of family it concerns, what could be possibly needed.” [interview 8, gynecologist]*.

What was specifically being discussed during counseling by the professionals at Youth Healthcare Centers was unclear to many participants, regardless of the participants’ awareness of any guidelines for counseling by their colleagues at these centers. However, they did not feel any urge to ask their regional Youth Healthcare facility for more information.

#### Views on maternal tdap vaccination

Participants generally had a positive attitude toward maternal Tdap vaccination. Most of them believed themselves to be aware of the vaccine’s purpose, its necessity, and its benefits as opposed to potential harms. After asking a secondary care midwife her opinion on maternal Tdap vaccination, she responded:

*“It’s about its [maternal Tdap vaccination’s] efficiency. Its effectiveness has been proven and we have decided to start offering this vaccination, just like neighboring countries. That is why I am in favor of it being possible.” [interview 9, secondary care midwife]*.

In addition to its safety and effectiveness, participants indicated that the reduced infant vaccination schedule was an important reason for pregnant women to accept Tdap vaccination. Infants of vaccinated mothers receive one fewer dose during their primary vaccination series and start the series one month later than infants of non-vaccinated mothers.

Although all gynecologists seemed in favor of maternal vaccination, some primary care midwives voiced doubts about immunization in general, including maternal Tdap vaccination. As maternal Tdap vaccination was often interpreted as a ‘novel’ vaccine, several participants were unaware of its established safety profile. A few said that vaccination during pregnancy seemed counter-intuitive:

*“The feeling that you should decline many things during your pregnancy – such as certain foods, et cetera – but you would allow someone to inject yourself a vaccine. That feels odd and that is also why some pregnant women don’t want this vaccination.” [interview 3, primary care midwife]*.

Participants had the impression that from the perspective of pregnant women, COVID-19 vaccination did not influence attitudes towards maternal vaccination or vaccine hesitancy. The uptake may have been reduced for a short while, since making an appointment for maternal vaccination during COVID-19 lockdown periods seemed difficult.

#### General versus tailored counseling

Most often, counseling by obstetric care providers consisted of a brief introduction to the vaccination and presentation of the NIP while handing over the information packet, perhaps accompanied by some arguments in favor of immunization. Only a few participants discussed arguments against vaccination. Pregnant women asked frequently what obtaining the vaccine entails and whether it is safe for their unborn child. Participants said they had to adjust their counseling to a woman’s need for knowledge, in order to deliver the appropriate information according to her awareness of the vaccine:

*“There is, of course, a group of women who already had it [Tdap vaccination] during [a previous] pregnancy, so you can get through that [counseling] a bit faster as they already consciously chose for it that time; and of course a group that has already heard or read about it, but did not receive it before; and a group that says they didn’t notice anything about it at all.” [interview 16, primary care midwife]*.

Participants felt the need to stay well-informed about maternal Tdap vaccination in order to improve their counseling. Being informed raised their confidence in counseling pregnant women, even to those who were reluctant to getting vaccinated. Some participants argued that the recommendations were “restricted to a generalized view” of pregnant women. More than the recommended time and effort was necessary for counseling pregnant women with a low socioeconomic status or a migration background.

*“I find it very difficult to inform people in case of a language barrier. They are often vulnerable pregnant women. A huge amount of information transfer is lost there.” [interview 9, secondary care midwife]*.

Time and effort was also necessary in the form of providing the vaccine at the hospital in case the pregnancy demanded medical attention, especially when longer-term hospital admission was required.

*“I often see people who are admitted relatively early throughout pregnancy with [medical] issues. They tend to stay hospitalized for a long time. They are often unable to make an appointment [for Tdap vaccination] at the counseling center [Youth Healthcare Center].” [interview 6, gynecologist]*.

#### Provider responsibilities in vaccine promotion

The implementation guidelines suggest that obstetric care providers should merely introduce maternal Tdap vaccination in a superficial manner, and persuasive strategies for vaccine promotion are intentionally omitted from information for providers. However, many of our participants argued that informing pregnant women is their primary responsibility as the initial care provider. At the same time, while they can promote the vaccination, the choice whether or not to take the vaccine ultimately remains that of pregnant women.

*“It is nowadays no longer the case that the doctor gives advice and that the pregnant woman blindly says ‘Well that’s a good idea, doctor, I’m going to do that.’ It just doesn’t work that way anymore. You have to eventually give patients the responsibility themselves.” [Interview 5, gynecologist]*.

Some participants indicated they counseled objectively, with no promotion of the vaccine, since they viewed their own opinion or attitude as irrelevant when it comes to the pregnant women’s decisions about getting vaccinated.

*“It’s not about what I think or what I do. I think if you look at the information and odds of vaccine implications objectively, then it’s easy to do it [getting vaccinated]. Though I can’t – when I am counseling someone whether or not to take the vaccine – I can’t tell them that I would take it.” [interview 4, primary care midwife]*.

Some participants would rather merely mention the maternal Tdap vaccination while providing the information packet to pregnant women, and only perform the bare minimum of what is recommended in the guidelines, because no financial compensation is available for the time spent by obstetric care providers on counseling. A financial compensation may, according to some, contribute to the quality of vaccine promotion. Nevertheless, they felt compelled to invest in information delivery due to the relationship they had acquired with their clients or patients.

*“I could also choose to only give the leaflet and say: “Go, find out what to do for yourself.” But I don’t think that is considered as providing sufficient care. It doesn’t work like that either.“ [interview 15, primary care midwife]*.

#### Impact of materials for information delivery

Participants said that the information letter and leaflet were appreciated by their pregnant patients; the illustrations and patient-friendly layout of the leaflet seemed to positively affect vaccination intention. They concluded however, that pregnant women received an overwhelming amount of information materials in the first trimester of pregnancy, and that Tdap vaccination materials might best be bundled with other materials to improve information delivery and information uptake by pregnant women. Participants also argued that handing over materials for vaccine promotion must always be supported by verbal information delivery.

*“Most pregnant women take it [information packet] and then it ends up at the bottom of the pile.” [interview 4, primary care midwife]*.

Participants mentioned occasionally that the information materials may be too difficult to understand by illiterate or non-Dutch-speaking pregnant women. The information materials were available in multiple languages, although hard copies were only available in Dutch. Therefore, the materials were less accessible, attractive and compelling to pregnant women with a migration background.

*“If they don’t speak the Dutch language, I will be forced to provide printed copies or send those to people by e-mail. It would be just useful if it’s all in such a shining leaflet [like the leaflet written in Dutch], so to speak. That you can give it right away [physically].” [interview 9, secondary care midwife]*.

## Discussion

This study indicated that the implementation of maternal Tdap vaccination in the Netherlands requires clear and transparent information about what the vaccination entails for obstetric care providers: what is expected from them, how they can obtain information, and when their actions must be initiated. Maternal Tdap vaccination was generally supported by obstetric care providers, mainly due to its proven effectiveness and its established safety profile. The participants were willing to invest time and effort in information delivery, even though the guidelines recommended only to make women aware of the vaccination, rather than counseling them extensively. As the pregnant women’s initial care provider, participants felt constrained to provide sufficient obstetric care.

Some participants argued that obstetric care providers were “kept in the dark” regarding the decision that Youth Healthcare physicians should administer the vaccine, as opposed to the obstetric care provider. Although there was close collaboration with Dutch professional obstetric organizations throughout the implementation process, it seemed that many participants were unaware of the opportunity for providing input via their umbrella organizations. In future implementations, the inclusion of care providers should be emphasized by such organizations, as research shows that involving providers during guideline development reduces resistance to recommendations [40, 41]. If professional organizations involve their members more actively, it may increase protocol adherence and improve attitudes toward vaccination among obstetric care providers, leading ultimately to higher vaccine uptake among pregnant women [23, 26, 42].

There was some contrasting between the different disciplines in obstetric care, predominantly between primary care midwives and gynecologists, with secondary care midwives in between. The debate on which party should facilitate vaccine administration, and whether or not this should be centralized, was a firm discussion between the disciplines that was already imbedded in the implementation. Even though it was occasionally interpreted as a ‘loss’ of the discussion as centralization was not realized, their attitude towards maternal vaccination seemed unaffected. Second, gynecologists had a generally more positive attitude towards maternal vaccination, compared to primary care midwives. This was in accordance with our previous questionnaire study [28]. Previous studies suggested that this is because gynecologists are better aware of the consequences, while among midwives, negative beliefs and concerns regarding vaccination, including vaccination in general, had risen [43, 44].

According to our participants, counseling of pregnant women about maternal Tdap vaccination cannot be standardized, as it highly depends on the women’s individual needs for knowledge. Moreover, some pregnant women required a more extensive approach, e.g. in a first pregnancy or a complicated pregnancy requiring extensive medical support. Our study emphasized that tailored counseling is needed for the provision of appropriate advice, making women feel their concerns have been addressed so they feel comforted about maternal vaccination [44–48]. Illiterate or non-Dutch speaking pregnant women are, according to our participants, more difficult to reach in terms of vaccine-promotion. To our knowledge, only one study assessed the influence of health literacy on maternal vaccine acceptance with higher literacy associated with rejection of the vaccine [49]. Nevertheless, the authors excluded women impacted by illiteracy and language barriers, which prevented completion of the questionnaires. High literacy was, however, associated with higher COVID-19 vaccine acceptance [50]. According to our findings, the first step to better target these groups in the Dutch maternal Tdap vaccination program could be facilitated by creating hard-copy information materials in multiple languages including making available a linguistic simplified version and by providing information about specific logistics for vaccine delivery when pregnancies demand medical attention.

Several participants who basically supported Tdap vaccination in pregnant women were hesitant to promote it; they described their counseling approach as objective or irrelevant, since women would ultimately decide for themselves. However, research has shown the relevance of provider attitudes; both verbal and non-verbal provider-patient communication greatly affect health-related outcomes, including vaccination intent among pregnant women [51]. Therefore, unwillingness to discuss or promote the vaccination could suggest to pregnant women that the provider has doubts about the vaccine. To improve care providers’ attitudes, we recommend that future implementation strategies facilitate providers’ needs and wishes, while also emphasizing that provider attitudes may subconsciously affect their counseling or its effect on pregnant women.

Our study has strengths and limitations. A strength of this study is that double coding was applied, thereby increasing the reliability of coding. As for the limitations, participants were selected from a pool of respondents to a prior questionnaire study, in which they indicated that they would like to participate in follow-up research. Therefore, only obstetric care providers who finished the questionnaire and provided their contact details could be included, possibly introducing selection bias [52]. Second, the interviews were conducted by researchers from the National Institute for Public Health, which is responsible for the implementation of the maternal Tdap vaccination. This link may have led some participants to speak less freely. On the other hand, some may have seen our evaluation as a unique opportunity to provide input for improvement of the implementation. In addition, the interviews were conducted by telephone due to the COVID-19 lockdown periods, which may have also contributed to hampered communication. Another limitation is that we asked participants for retrospective views on the implementation strategy and informational materials a full year after NIP included the vaccination; thus their recall may have been compromised. To reduce any other influence of recall bias, we started each interview by summarizing the different timepoints of the maternal Tdap implementation process and the date at which the vaccine was included within the NIP.

In conclusion, this study underlined the importance of involving the relevant healthcare professions, including individual care providers, during the implementation of a maternal Tdap vaccination in the NIP. Our participants generally supported the vaccination, but some were hesitant, especially about vaccination during pregnancy. Future implementation strategies involving antenatal care should focus on tailored information for pregnant women as opposed to generalized information that applies only to uncomplicated pregnancies.

## Electronic supplementary material

Below is the link to the electronic supplementary material.

Supplementary Material 1

## Data Availability

The datasets generated and analyzed during the current study are not publicly available due to linguistical barriers in the process of our study procedures, but are available from the corresponding author upon a reasonable request.

## Abbreviations

GP: General practitioner
KNOV: Royal Dutch Organization of Midwives
NIP: National Immunization Program
RIVM: National Institute for Public Health and the Environment
Tdap: Tetanus, diphtheria and acellular pertussis
