# Supplementary material for: Maternal vaccination against pertussis as part of the national immunization program: a qualitative evaluation among obstetric care providers one year after the implementation in December 2019
Source: BMC Health Serv Res. 2023 Mar 30;23:311. doi: 10.1186/s12913-023-09274-1 (PMC10062680; doi:10.1186/s12913-023-09274-1)
Supplement: Supplementary file 1 — Supplementary Material 1 [file 12913_2023_9274_MOESM1_ESM.docx]

**Supplementary Table.** The five major themes with corresponding categories, subcategories and example codes.

| Theme | Category | Subcategory | Example code^a^ (translated from Dutch) |
| --- | --- | --- | --- |
| 1) Challenges throughout the implementation process | General process | Organization | *one must decide when the vaccination will be implemented* |
|  |  | Training session(s) | *keep information provision this way* |
|  |  | Naming the vaccine | *name 22-week-shot^b^ follows up the 20 weeks anomaly scan quite well* |
|  |  | Time lapse since advice | *inform care providers directly after advice of health council* |
|  |  | Logistics pre-inclusion | *implementation was picked up together with GPs* |
|  |  | Logistics post-inclusion | *messy start due to uncertainty on logistics* |
|  |  | Execution | *execution became easier later on* |
|  |  | Vaccine stock | *vaccines were not always available right after implementation* |
|  | Allocation of roles | Current guidelines | *in doubt whether the vaccine is currently administered at the right place* |
|  |  | Centralization | *the whole process needs to be put in one place* |
|  | Collaborations | Contact with Youth Healthcare Centers | *contact is good, often concerns other things than maternal vaccination* |
|  |  | Content of consult at Youth Healthcare Centers | *unaware how they [youth healthcare professionals] counsel* |
|  |  | Post-partum care | *they initiate post-partum care during pregnancy* |
|  |  | Availability | *contacting them takes some effort sometimes* |
| 2) Views on maternal Tdap vaccination | Attitude towards maternal vaccination | Efficiency | *maternal pertussis vaccination has been proven to be efficient* |
|  |  | Safety | *unavailable long-term effects of maternal vaccination* |
|  |  | Necessity | *maternal pertussis vaccination prevents serious complications in their infant* |
|  |  | Disease severity | *whooping cough may lead to death* |
|  |  | Benefits of vaccination | *vaccination has a twofold strategy [prevention of disease and infant fewer vaccinations]* |
|  |  | Drawbacks of vaccination | *odd feeling to have a vaccination injected during pregnancy* |
|  |  | Vaccine components | *not okay that the vaccine is a cocktail [of vaccine components]* |
|  | Attitude towards general vaccination | Infant vaccination | *that their baby may be vaccinated a month later is an incentive for women to accept the vaccination* |
|  |  | Experiences with own children | *own children did not receive all vaccinations as well* |
|  |  | COVID-19 vaccination | *during the corona time making an appointment [for maternal vaccination] was difficult for a while* |
| 3) General versus tailored counseling | Consultation | Information provision and referral | *mentions the existence of the vaccination* |
|  |  | Timing during pregnancy | *the intake [first appointment] is too early for [explaining] the maternal vaccination* |
|  |  | Acceptance or rejection | *some women have principles against vaccination* |
|  |  | Time and effort | *sometimes a challenge to discuss everything in addition to other things* |
|  |  | Documentation in medical files | *women are asked whether or not they obtained the vaccination, which will be documented* |
|  |  | Need for knowledge | *opinion on vaccinations differs between women* |
|  | Pregnant women’s situation | Primi- or multiparous women | *pregnant women compare the situation to their first pregnancy* |
|  |  | Socio-economic status | *there are many socially vulnerable pregnant women* |
|  |  | Hospitalization | *insufficient information [available] for hospitalized pregnant women* |
| 4) Provider responsibilities in vaccine promotion | Job assignments | Self-perceived assignments | *my job is to educate women and to tell them the benefits comprehensively* |
|  |  | Objective counseling | *the opinion of the care provider [on vaccination] does not matter* |
|  |  | Persuasive approach | *care provider must deduct barriers and reduce thresholds* |
|  |  | Ultimate decision | *give patients responsibility to decide themselves* |
|  |  | Financial compensation | *all those extra tasks for midwives are not financially justified* |
|  |  | Doubt whether or not to answer questions | *we [obstetric care providers] will be asked questions [instead of Youth Healthcare professionals]* |
|  | Pregnant women’s perspectives | Opinion on immunization (during pregnancy) | *different conversations with vaccine-critical women* |
|  |  | Acquired relationship with care provider | *bond between midwife and pregnant forces midwife to counsel about maternal vaccination* |
| 5) Impact of materials for information delivery | Mandated materials | Clarity – difficulty | *current materials for information are structured* |
|  |  | Size and scope | *bundled materials should keep it manageable* |
|  |  | Linguistics | *language barriers result into loss of information transfer* |
|  |  | Literacy | *extra attention required for illiterate people* |
|  | Self-composed additional materials | (Digital) mail, link to official website [1] | *redirect women to the website for additional information* |
|  |  | Other materials | *those thematic pink bandages are a good image for the vaccination* |

^a^ Example codes reflected solely the code, and not the marked segments from the transcripts. Quotations have been provided in the Results section of this article. ^b^ Official name for the maternal Tdap vaccination [in Dutch: 22-wekenprik]

[1] 22-wekenprik. Rijksinstituut voor Volksgezondheid en Milieu (RIVM); 2019.
